# Mango Peel Pectin: Recovery, Functionality and Sustainable Uses

**DOI:** 10.3390/polym13223898

**Published:** 2021-11-11

**Authors:** Malaiporn Wongkaew, Pikulthong Chaimongkol, Noppol Leksawasdi, Kittisak Jantanasakulwong, Pornchai Rachtanapun, Phisit Seesuriyachan, Yuthana Phimolsiripol, Thanongsak Chaiyaso, Warintorn Ruksiriwanich, Pensak Jantrawut, Sarana Rose Sommano

**Affiliations:** 1 Program in Food Production and Innovation, College of Integrated Science and Technology, Rajamangala University of Technology Lanna, Chiang Mai 50220, Thailand; malaiporn@rmutl.ac.th; 2 Plant Bioactive Compound Laboratory, Faculty of Agriculture, Chiang Mai University, Chiang Mai 50200, Thailand; 3 Program in General Education, College of Integrated Science and Technology, Rajamangala University of Technology Lanna, Chiang Mai 50220, Thailand; c.pikulthong@rmutl.ac.th; 4 School of Agro-Industry, Faculty of Agro-Industry, Chiang Mai University, Chiang Mai 50100, Thailand; noppol@hotmail.com (N.L.); jantanasakulwong.k@gmail.com (K.J.); pornchai.r@cmu.ac.th (P.R.); phisit.s@cmu.ac.th (P.S.); yuthana.p@cmu.ac.th (Y.P.); thanongsak.c@cmu.ac.th (T.C.); 5 Cluster of Agro Bio-Circular-Green Industry (Agro BCG), Chiang Mai University, Chiang Mai 50100, Thailand; 6 Department of Pharmaceutical Sciences, Faculty of Pharmacy, Chiang Mai University, Chiang Mai 50200, Thailand; warintorn.ruksiri@cmu.ac.th (W.R.); pensak.j@cmu.ac.th (P.J.); 7 Cluster of Research and Development of Pharmaceutical and Natural Products Innovation for Human or Animal, Chiang Mai University, Chiang Mai 50200, Thailand

**Keywords:** extraction technique, fruit characteristic, mango peel biorefinery, pectic polysaccharide, pectin source

## Abstract

Concerns regarding the overconsumption of natural resources has provoked the recovery of biopolymers from food processing biomass. Furthermore, the current market opportunity for pectin in other areas has increased, necessitating the search for alternative pectin resources. This is also a step towards the sustainable and circular green economy. Mango peel is the byproduct of agro-processing and has been used for high value-added components such as polysaccharide biopolymers. Pectin derived from the peel is yet to be exploited to its greatest extent, particularly in terms of its separation and physiochemical properties, which limit its applicability to dietary fiber in culinary applications. The functionality of the mango peel pectin (MPP) strongly depends on the molecular size and degree of esterification which highlight the importance of isolation and characterisation of pectin from this novel resource. This article therefore provides a useful overview of mango peel as a potential biomaterial for the recovery of MPP. Different extraction techniques and the integrated recovery were also discussed. The utilisation of MPP in different industrial schemes are also detailed out from different perspectives such as the pharmaceutical and biotechnology industries. This review convincingly expresses the significance of MPP, providing a sustainable opportunity for food and pharmaceutical development.

## 1. Introduction

Fruits are widely used in agri-food industry in which large quantity of by-products including pomace, peel, rind and seeds are generated [1,2]. This biomass is a potential source for valuable bioactive compound recovery such as dietary fibres, carotenoids, polyphenols, oils, vitamins and many other compounds [3]. Mango is one of the most consumed tropical fruits, known for its high nutritive values and extensively cultivated in the tropical and sub-tropical regions. Several preserved products of mango are commercially needed such as can, dried mango, frozen slices, purée, juices and nectar [4,5,6]. It is estimated that around 200,000 tons of biomass are generated during these processing and peels account for as high as 25% of the volume [7]. So far, attempts have been made in trying to value-add such the biomass from mango processing using integrated refinery approaches [8,9,10,11,12,13]. Besides its high contents of carbohydrates, proteins, fats and various classes antioxidants such as polyphenols, carotenoids and vitamins [14,15,16,17], this high-volume biomass is known as a potential source of dietary fibre [10,18,19,20]. The soluble dietary fibre is a carbohydrate polymer with more than 10 monomeric units that makes it is difficult to be hydrolysed by endogenous enzymes in the human small intestine [21,22]. They include pectin, galactomannan, inulin, gum while pectin is of high commercial need for functional foods and pharmaceutical applications [23,24,25,26]. Besides, mango peel contains high cellulose content (30%) and lignin (16%) [27,28]. As a result, it was employed as a novel source for biopolymer recovery. Additionally, it comprises of 5–20% of pectin with variable contents of galacturonic acid, dependent upon the extraction methods and the cultivars [10,18,19,20]. To extract such the value-added biopolymers, the integrated isolation approach can be used [29].

The global need for pectin as biopolymer amounted to $1 billion in 2019 and is expected to rise to $1.5 billion in 2025 [26]. Commercial pectin is mainly recovered from either apple pomace or citrus peel which are of different physicochemical functionalities based up on the presence of pectin esterase of the raw materials [30]. Apple pomace pectin forms a gel of high viscosity which is suitable as a medicinal polymer, while the lighter colour of citrus pectin is preferable in the confectionery industry. Biopolymer pectin for industry requires a minimum of 65% of galacturonic acid on ash and moisture-free substances which limit other potential new resources for pectin recovery. In recent years, the recovery of non-starch polysaccharides from fruit by-products has become a promising strategy for the development of natural biopolymers [31]. Besides these, the information on mango peel pectin (MPP) as a potential biopolymer for industrial applications is not collective. In this study, the characteristics, value adding components and biorefinery process of mango peel are discussed. Featuring the most-sought after pectin biopolymer, its chemical structures and different extraction processes are highlighted along with possible applications in different industries are collectively presented. This review provides a useful baseline for substantial production of MPP as well as a guidance for the global policy of zero-waste processing and sustainable used of natural resource.

## 2. Mango Peel as the Novel Source for Pectin Biopolymer

### 2.1. Mango Variety

A wide range of mango varieties are cultivated including those of native and new bred cultivars. Therefore, the yield and the physiological attributes are diverse, depending on their gene pools and further interaction with environmental conditions [32,33]. Physical characteristics such as fruit weight, size and peel colour have been used to describe mango varieties. These physical attributes also play a crucial role in consumption and industrial processing. The commercial attributes of mango physiology are illustrated in Table 1. The CIE colour space (L*, a*, b*) has been used to determine maturity index and the ripening process of mangoes [34,35]. The information of length, width and breadth of mango fruits are used for arithmetic mean diameter (D_a_) and geometric mean diameter [36] data calculation. These values are regarded as physical parameters during fruit grading [37]. Likewise, the ratio of width-to-length or aspect ratio (R_a_) indicates an ellipsoid shape during the process of fruit development [38]. The greater value of R_a_ signifies more advanced ripening stages of the fruits [39]. In addition, specific gravity as defined as fruit soluble matters of the sugar contents along with firmness alteration can be typical used to define the stage of maturity [5]. It is worth highlighting that both parameters intercorrelate with each other, and greater values of R_a_ and sphericity denote an advanced stages of fruit ripening [39]. Moreover, a higher fruit ripeness leads to a greater content of pectin from fruit peel [40]. Wongkaew et al. [6] reported that the physical properties of fruit physical properties (Colour, D_a_, D_g_, R_a_, sphericity, surface area and percentage yield of fruit parts) can be used to distinguish mango varieties.

**Figure 1:**
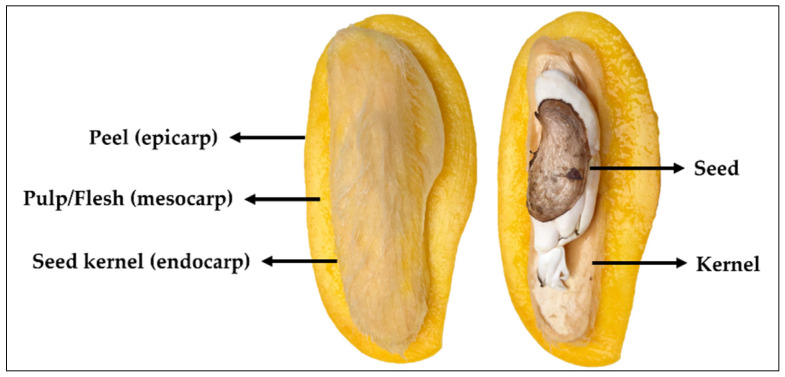
The biomass composition of mango fruit.

The morphology of mango fruit comprises of three parts, namely pulp (mesocarp), peel (epicarp) and seed kernel (endocarp), as illustrated in Figure 1. Mango pulp is a source of a variety of phytochemical components including those of reducing sugars, amino acids, aromatic compounds as well as functionally active ingredients, such as pectin, vitamins, anthocyanins and polyphenols [41]. In processing, pulp is the most-consumed parts of the fruit, while the peel and seed are usually discarded (accounted for 35–60% of the total fruit weight) as biomass [7]. Peel (~5–17%) and seed (~7–17%) are known as byproducts of mango processing and the amount depends on mango varieties. These are, indeed, the potential resource for natural product biorefinery.

**Table 1:** Physical characteristics of different ripe mango varieties.

Mango Varieties	Colour			Length (mm)	Width (mm)	Breadth (mm)	Volume (mL)	Flesh Weight (%)	Peel Weight (%)	Seed Weight (%)	References
	L*	a*	b*								
Mahachanok	68.83	3.28	40.66	165.05 **	66.42 **	58.30 **	313.89 **	66.69	16.64	16.66	[6]
Chok Anan	69.98	5.55	43.09	111.77 **	74.80 **	63.1 **	217.49 **	67.32	14.32	18.29	
Nam Dok Mai	72.26	6.74	36.63	140.14 **	70.61 **	62.10 **	271.47 **	73.15	14.42	12.43	
Kaew	67.68	3.41	39.70	112.16 **	70.66 **	62.45 **	209.54 **	70.32	15.60	14.08	
Rad	47.19	0.26	17.03	98.47	51.67	45.89	n/a	n/a	5.40	n/a	[13]
Ta Labnak	33.09	−6.01	9.51	87.26	81.47	69.17	n/a	n/a	6.00	n/a	
Sampee	47.43	3.28	20.36	93.62	55.69	47.65	n/a	n/a	7.60	n/a	
Nyala	n/a	n/a	n/a	83.00	83.00	n/a	150.00	82.10	10.70	7.20	[42]
Edelfursan	n/a	n/a	n/a	92.00	92.00	n/a	250.00	81.57	10.53	7.90	
Kaboom	n/a	n/a	n/a	95.00	95.00	n/a	300.00	82.40	10.60	7.00	
Alphonso	n/a	n/a	n/a	94.60	73.40	60.60	214.40	74.58	14.19	11.22	[43]
Kesar	n/a	n/a	n/a	95.70	65.70	58.00	188.80	71.28	13.06	15.66	
Totapuri	n/a	n/a	n/a	123.60	70.80	66.60	261.50	71.33	16.42	12.25	

** unpublished data by the first author; n/a = not available.

### 2.2. Value-Added Components from Mango Peel

As mentioned, peel and seed are the major by-products of the mango processing industry. These biomasses are usually buried in landfill or used as animal feed that; the fermentation process is generally toxic to the soil [44]. Previous reports claimed that mango peel consists of various valuable phytochemicals, including pectin, carotenoids, polyphenols and other bioactive compounds that can be used in the pharmaceutical industry [8,17,22,45]. However, these compositions are variable depending on the maturity stage, locality, variety and climatic conditions where mangoes are produced.

As presented in Table 2, mango peel contains a variety of macronutrients viz., carbohydrates, protein, lipid and crude fibre. Crude fibre is an important element to determine the significance of the by-products as sources of pectin recovery. In mango peel, the fibre content ranges between 2–20% of the total mass. While citrus peels contain as high as (>50%) dietary fibre and different varieties are currently used as raw material for citrus peel pectin including those from Valencia orange [46], Persia lime [46], lemon [47] and sweet orange [47]. Owing to its high fibre content, the peel can be used as an additive ingredient to enhance the functional properties of food and feed [48,49]. Calcium is the largest mineral constituent in the peel followed by magnesium, potassium and sodium, respectively [7]. The content of vitamin C ranges from 18–257 mg·g^−1^, depending again on the varieties. The vitamin E of ripened mango peel is of a greater concentration than the green peel [41].

The polyphenol content in the peel varies from 55–110 mg·g^−1^ dry weight and higher levels are found in the ripe fruit than they are in the unripe peel [16]. The peel is also the major source of polyphenols that are basically higher than the pulp at all growth stages [50,51]. Mangiferin (C-glucosyl xanthone), a heat-stable and pharmacologically active phytochemical, is typically found in high content of mango peel. Mangiferin illustrates various bioactivities such as antiinflammation, anti-diabetic, immunomodulatory, antitumor and antioxidant [52]. The amount of mangiferin and its derivatives is greater in the peel than in the pulp [53]. As a result of its functional properties, mangiferin is commercially used in term of therapeutic and cosmetics products [54] and food supplements [55].

Anthocyanins, water-soluble pigments, add red, blue and purple colours to the peels of mangoes. The compounds are known for their beneficial effects in the prevention of various diseases such as cancer, diabetes and neuronal and cardiovascular diseases, thereby promoting human health [56,57]. Total anthocyanin content ranges from 3600–5650 µg·g^−1^ in the fully ripe stage and from 2030–3260 µg·g^−1^ in the raw and unripe stages [16]. The major anthocyanins detected in various varieties of mangoes, namely cyanidin, pelargonidin, delphinidin, malvidin, petunidin and peonidin [58]. Regarding to their biological properties, anthocyanins are comprehensively used as a substitute for artificial colorants in foods and beverages [59,60].

Carotenoids are fat-soluble pigments that give peels and flesh their yellow, orange and red colours. Mango peel contains high concentrations of carotenoids in the form of β-carotene, a precursor for vitamin A [61]. The content of carotenoids generally increases during ripening and is the highest at the fully-ripe stage [58]. Consumption of carotenoids reduces the risk of developing certain cancers (cervical, ovarian, colorectal, prostate, breast), cardiovascular disease, bone, skin, or eye disorders, mental health, metabolic health, during pregnancy and early life and even provide cosmetic benefits [62,63]. As a result, carotenoids are also widely used in food as a colourant, antioxidant and additive [64].

**Table 2:** Nutritional and phytochemical compositions of mango peel [6,13,41,49,65,66,67,68,69].

Compounds	Content
Macronutrients (%)	
Water	31.30–76.70
Carbohydrate	10.53–30.80
Protein	2.10–8.06
Total lipid	1.40–2.48
Total sugar	25.00
Total dietary fibre	1.40–20.53
Minerals (mg·100 g^−1^)	
Calcium	150
Iron	40.6
Magnesium	100
Potassium	75
Sodium	50
Copper	10.4
Vitamins	
Vitamin C (total ascorbic acid, mg·100 g^−1^)	18–257
Vitamin A (retinol activity equivalent, μg ·100 g^−1^)	100
Vitamin E (α-tocopherol, mg·100 g^−1^)	0.25–0.59
Polyphenols (mg·100 g^−1^)	
Kaempferol	3.6
Mangiferin	169
Mangiferin gallate	321
Isomangiferin	13.4
Quercetin	6.5
Rhamnetin 3-0 galactoside/glucoside	9.4
Flavonoids (catechin equivalent·100 g^−1^)	
Anthocyanins (μg)	3600–5650
Cyanidin	22.10
Pelargonidins	22.73
Delphinidins	18.02
Malvidins	5.26
Petunidins	21.60
Peonidins	24.42
Carotenoids (μg)	3092
β-carotene	1310
β-cryptoxanthin	600
Lutein and zeaxanthin	299

### 2.3. High Value-Added Components Biorefinery

Table 3 details out research studies on the phytochemical biorefinery of mango by-product, mainly peel. The biorefinery not only value-adds the biomass but also reduces the biomass volume from the industrial processing of mango. Owing to a disposal of the loss, transportation costs and limited availability of landfills are questionable for sustainable processing. Thereby, mango peel valorisation through different techniques would undoubtedly eliminate the disposal problem.

## 3. Mango Peel Pectin

### 3.1. Mango Peel Pectin Recovery

General pectin recovery includes a raw material pre-treatment stage, an extraction operation and a post-extraction stage [86,87]. Nevertheless, the issue on the conventional process, particularly the extraction step, is whether or not it is worth the energy and economic demands that are currently required in the practice [88]. Therefore, several sustainable and quicker alternative approaches to extract pectin from biological materials have been developed. The innovative techniques for pectin extraction include enzyme-assisted extraction, ultrasounds, subcritical fluids and microwave heating. The benefits and drawbacks of the techniques are compared as shown in Table 4.

#### 3.1.1. Conventional Heating Extraction (CHE)

Pectin is traditionally extracted in water acidified with 0.05–2 M sulfuric, nitric, phosphoric, acetic or hydrochloric acid between 80–100 °C for 1 h with continuous stirring [89]. Conventional extraction (solid–liquid extraction) depends on a number of factors such as temperature, pH, solvent properties, solid to solvent ratio, dry solids, particle size and diffusion rate [90]. For pectin extraction, mango peel powder was initially treated with the acidified solution. Subsequently, the obtained solvent was treated with ethanol solution [91]. Through this method, an MPP yield as high as 30% can be achieved from the residue with the degree of esterification (DE) varying from approximately 60 to 90% [10,91].

#### 3.1.2. Novel Extraction Techniques

- Microwave-Assisted Extraction (MAE)

MAE involves dielectric heating of plant molecules through the exposure of microwaves. The microwave irradiation accelerates cell rupture by a sudden temperature rise and internal pressure increase inside the cells of plant sample, which promotes the destruction of sample surface and in turns the exudation of pectin within the plant cells into the surrounding solvents and increase [92,93,94]. The conventional “on-off” microwave operation, however, may lead to the overheating of the raw material, which may ultimately result in a low quality of MPP. Consequently, a phase controller (PCMAE), which regulates the electrical power input into the magnetron thereby generating smooth and adjustable microwave power was installed additionally for a better extraction performance [10]. The applications of the MAE for pectin extraction from mango peel were reported and the obtained pectin had higher content when compared with the CHE [10,12]. The microwave provides more efficient heat than the CHE approach due to the intense formation of vapour in polar substances generated by the electromagnetic field [95].

- Enzyme-Assisted Extraction (EAE)

The enzymes are used to improve extraction process by hydrolyzing matrix of the plant cell wall. Cell wall degrading enzymes with minimum pectinolytic activity are used to hydrolyze non-pectin plant cell wall components in enzymatic extraction of pectin [96,97]. The EAE depends on reaction time, type and concentration of enzyme, temperature, pH value and particle size of plant material [98,99]. The EAE technique was applied to recover pectin from multiple bioresources such as lime [100], passion fruit [101] and apple pomace [102]. The yields of pectin were achieved with the enzymatic extraction which were greater than that obtained with the CHE method. However, the pectin extraction from mango peel using this technology has not yet been implemented.

- Ultrasound-Assisted Extraction (UAE)

Sound waves consist of mechanical vibrations, which can be applied in treatments to the solid, liquid or gas with frequencies higher than 20 kHz [99,103]. Adapted for pectin extraction, the collapse of cavitation bubbles near cell walls induced by ultrasound produces cell disruption, thus causing stronger and enhanced solvent entrance into the cells and intensification of the mass transfer [104,105]. For pectin recovery, Guandalini et al. [106] found that the UAE provided an alternative choice for pectin extraction from mango peel because through this technique an MPP yield as high as 50% can be achieved without interfering the physicochemical properties (galacturonic acid content and degree of esterification).

- Subcritical-Assisted Extraction (SWE)

Subcritical water is liquid water at elevated pressure which is able to attain temperatures higher than its normal boiling point without a change in phase. When such water is used as solvent in extraction, the process is known as subcritical water extraction (SWE) also known as pressurized hot water extraction (PHWE) and superheated water extraction (SHWE) [107]. The SWE is stated as a green route for the valorisation of mango peel in form of pectin product. Xiaa and Matharu [108] reported that the MPP extracted by the SWE with no mineral acid supplementation resulted in a great yield of 18.34%, while the DE of the pectin was more than 70%.

**Table 4:** Benefits and drawbacks of the novel techniques.

Extraction Techniques	Benefits	Drawbacks
MAE	- Reduce extraction time - Low solvent requirement - Improve the quality and quantity of pectin than conventional technique - Considered as green technology [109,110]	- Corrosion problem on equipment from acidified water used [111]
EAE	- No use of acidic pH levels and high temperature - No corrosion problem on equipment - Consider as green technology - Reduce need for certain pre-treatment steps - Decrease in overall extraction time and a faster extraction process - Improve the pectin quality due to the mild condition of extraction [112,113,114]	- High cost of enzyme - Scale-up of EAE process can be difficult because of the uniqueness in response of different enzymes to changing environmental conditions [97]
UAE	- Reduce extraction time, reduced energy consumption and a relatively lower use of solvent - Enhance the yield and kinetics of pectin during recovery is biomass-specific plant [111,115]	- Does not greatly reduce solvent requirement after all [116]
SWE	- High quality extracts - Quick extraction process - Save in solvent use (water) and - making this technique suitable for food and pharmaceutical compounds [107,117]	- High cost of technique implementation - Pectin degradation [118]

### 3.2. MPP Functionality

Pectin is mostly extracted from various plant sources and is of great variation in term of quality. Consequently, pectin is purified and restructured in order to achieve constant and reproducible gel strength, for example HMP is improved its quality by dilution with sucrose. MPP is typical of high methoxyl content which is unable to form gel by interaction with calcium ions due to an insufficient number of carboxylic groups [8,119]. Thus, to improve its functionality for a specific purpose, de-esterification using either acidic or basic chemicals is necessary. The characteristic compositions of the extracted MPP are illustrated in Table 5.

**Figure 2:**
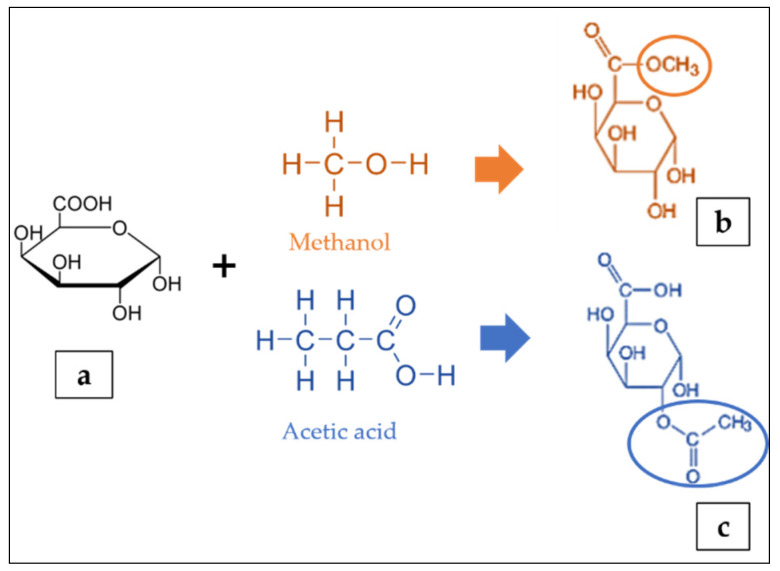
Structure of galacturonic acid (**a**) presenting methyl esterified (**b**) and acetylated (**c**) forms adapted from [122].

The residues of galacturonic acid (GA) (Figure 2a) are generally recognised as the backbone of the pectin structure. Its chemical structure composes of an aldehyde group at C1 and a carboxylic acid group at C6 [25]. The GA can be partially methyl-esterified at C6 with methanol and acetylated at the O2 or O3 positions with acetic acid (Figure 2b,c) [122]. The GA content can be determined by either the colorimetry [106] or high performance liquid chromatography [123]. The ratio of methyl-esterified galacturonic acid groups to the total galacturonic acid groups is defined as the degree of esterification (DE) [124,125,126]. The degree of esterification and acetylation of pectin affects the gelling properties of the pectin; a higher DE increases the capacity to form gels, whereas a higher degree of acetylation inhibits gelling [127]. The analytical quantification of DE include the titrimetric technique [106,128], gas liquid chromatography and colorimetric uronic acid analyses [129]. Furthermore, the content of GA in foods is very important because their presence can affect the chemical and sensorial characteristics of the matrix such as pH, total acidity, microbial stability, sweetness, consumer acceptability and therefore, provide precious information on the wholesome quality of the food or on the optimisation needed to impart select technical features [130]. Meanwhile, the molecular weight of pectin depends on the raw materials and the extraction techniques. Bagherian et al. [109] reported that continued heating of pectin extraction may lead to pectin networks disaggregation, thus decreasing the molecular weight.

In case of pectin recovered from mango peel, the GalA contents varied depending on the extraction techniques. Process optimization of extraction methods to obtain the minimal GalA level of 65% in MPP has been highlighted in many research studies [121,131,132]. Geerkens et al. [133] claimed that the preparation processes of the peel (blanching, particle size reduction) and fruit ripening stage reduced the GalA content, however the highest content obtained was 48%. Regarding the DE content, the values were in a range between 56% and 93%, which categorized it as high methoxyl pectin [134]. Both GalA and DE of pectic polysaccharides are involved in the commercial uses of pectin as gelling and thickening agents [135,136].

### 3.3. MPP Applications

Pectins are widely used as additive in foods and beverages such as a gelling agent, thickener, texturiser, emulsifier and stabiliser [137]. In recent years, pectin has been applied as a fat or sugar alternative in low-calorie foods [12], dietetic food [138], food packaging [139] and drug carrier [119]. Selection of pectin for a particular food depends on many factors, including the texture required, pH, processing temperature, presence of ions, proteins and the expected shelf life of the product [140]. MPP was recovered from peel of ‘Nam dok mai’ variety (Mox > 8%) and was found suitable as fat replacement in a Chinese sausage formular in its original form and colour [12]. Additionally, MPP obtained from ‘Chok anan’ variety was utilised as a substrate for pectic oligosaccharide hydrolysate with pectinase. The digested monosaccharide compositions were mainly fructose and glucose while arabinose had prominent influence on prebiotic potentials of *Bifidobacterium animalis* [141]. Thin films have been used as food packaging polymer and many drug delivery systems of oral, buccal, and transdermal routes. In one study, thin film was fabricated from a mixture of LMP and MPP at 1:2 ratio with 40% (*w/w*) glycerol. The film attained the highest elongation at break (8.80%) and lowest Young’s modulus (83.19 MPa) with an increasing hydrophobicity when the content of MPP increased [8]. For a topical drug delivery, de-esterified MPP with NaOH was proposed for thin film development [119]. In this same study, the DE decreased when a higher volume (~3.0 mL) of 1 N NaOH at 25 °C was employed in the preparation.

**Figure 3:**
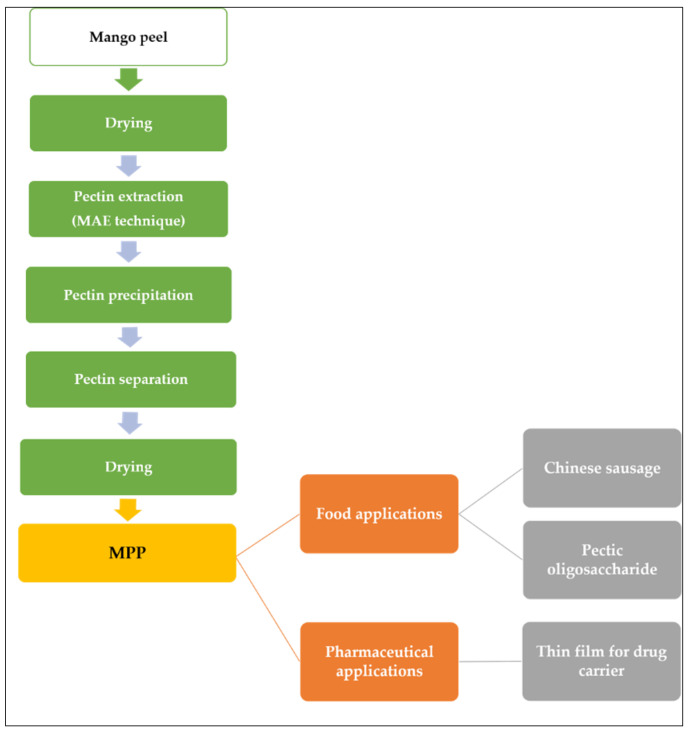
MPP value chain and applications.

Wongkaew et al. [6] explained the industrial value chain process of MPP as illustrated in Figure 3. First, the biomass was dried and pectin extraction can be achieved with MAE techniques. The dried peel powder was suspended in diluted acidic solution (distilled H_2_O adjusted to pH 1.5 with 2 M HCl) and heated in a microwave oven followed by separating the residue from the solution using filtration technique. The liquid is combined with a 1:1 ethanol-water mixture to precipitate the pectin, and then it is separated by filtration. The pectin was dried at 40 °C until a consistent weight was attained. The final product can be applied to food additives or sources of prebiotic or in pharmaceutical application.

### 3.4. Future Direction of MPP Utilisation

**Figure 4:**
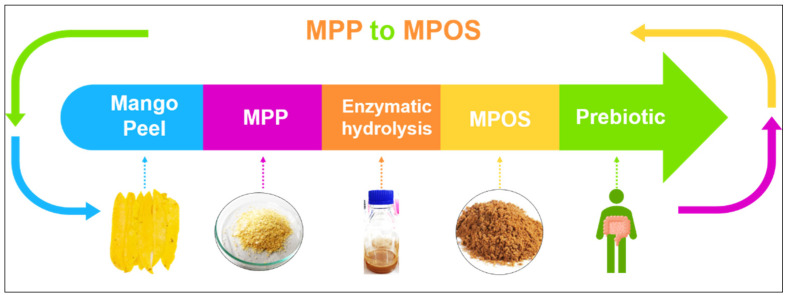
Future direction of MPP utilisation to MPOS production.

Plant polysaccharides are vital for the modulation of human gut microbiota which can impact on health generally recognised as prebiotics [142]. Among the most common prebiotic candidates, pectin oligosaccharide (POS) is receiving attention in the functional food industry [143]. MPP can possibly be hydrolysed into small molecules of pectic oligosaccharide or MPOS, as shown in Figure 4 [144]. The MPOS obtained highly stimulated the probiotic growth as well as the total short-chain fatty acids (SCFAs) production of *Bifidobacterium animalis* TISTR 2195 and *Lactobacillus reuteri* DSM 17938. It is also confirmed in our previous study that the MPOS illustrates a high potential as a prebiotic property [141]. The subsequently obtained SCFAs provide a great variety of health effects, including inhibition of pathogenic bacteria, constipation relief, reduction in blood glucose levels, improvement in mineral absorption, reduction of colonic cancer and modulation of the immune system [145].

## 4. Conclusions

Peels account for around a quarter of the entire mango fruit which is generated during the large-scale processing. Mango peel pectin can be retrieved from this biomass and its functionality depends upon the physiochemical characteristics which are largely influenced by varieties and extraction techniques. The high methoxyl content of the recovered pectin limits its use as food additive only. This biopolymer is structurally conversed by the de-esterification with alkaline treatment, resulting in its extended use as packaging or pharmaceutical-drug carrier. Future direction is heading toward the use of this potential biopolymer as functional prebiotic ingredient. This review pulls together the landscape picture of mango peel pectin biopolymer and highlights the use of the biomass as alternative biorefinery material to encourage a global sustainable development approach.

## Figures and Tables

**Table 3:** Current research studies on mango peel biorefinery of various value-add products.

Biorefinery Aspects	Products	References
Biotechnological aspects	Ethanol production	[27,70]
	Wine fermentation	[71]
	Enzyme production ● Carboxymethyl cellulase ● Cellulase ● Pectinase	[72,73,74]
	Lactic acid production	[75]
	Single cell protein production	[76]
	Sugar source	[77]
Sources for functional ingredients	Pectin	[6,10,12,13]
	Phenolic compounds	[78,79,80]
	Carotenoid	[16,71]
	Functional food ingredient ● Noodles, bread, biscuits, sponge cakes, other bakery products and yogurt	[81,82]
	Dietary fibre ● Macaroni ● Beef burger	[83] [78]
Other application areas	Removal of heavy metals	[84]
	Pharmaceutical excipient	[20,85]

**Table 5:** Typical characteristics of mango peel pectin compared with commercial pectin.

Characteristics	Commercial Pectin [120]	Mango Peel Pectin		
		CHE [106]	UAE [121]	MAE [6]
Galacturonic acid (%)	>65 (typically 75–80)	76	52–53	n/a
Degree of esterification (%)	30–75	61	56–93	57–93
Degree of acethylation	<5 (except for e.g., sugar beet pectin)	n/a	n/a	n/a
Neutral sugars (%)	<15%	n/a	n/a	n/a
Protein (N × 6.25) (%)	<5%	n/a	4.7–5.9	n/a
Molecular weight (g mol^−1^)	100,000–200,000	n/a	378,400–2,858,000	n/a

n/a = not available; CHE = conventional heating extraction; UAE = ultrasound-assisted extraction; MAE = microwave-assisted extraction.

## Data Availability

Not applicable.
